# Supplementary material for: ﻿Discovery of a new tarantula species from the Madrean Sky Islands and the first documented instance of syntopy between two montane endemics (Araneae, Theraphosidae, Aphonopelma): a case of prior mistaken identity
Source: Zookeys. 2024 Aug 16;1210:61–98. doi: 10.3897/zookeys.1210.125318 (PMC11344175; doi:10.3897/zookeys.1210.125318)
Supplement: Supplementary material 5 — Boxplots comparing various morphometric ratios based on measurements of mature male and female members of the Marxi species group [file zookeys-1210-061_article-125318__-s005.zip › Suppl5/Marxi_group_males/PDFs/T3P4_ratio_males.pdf]

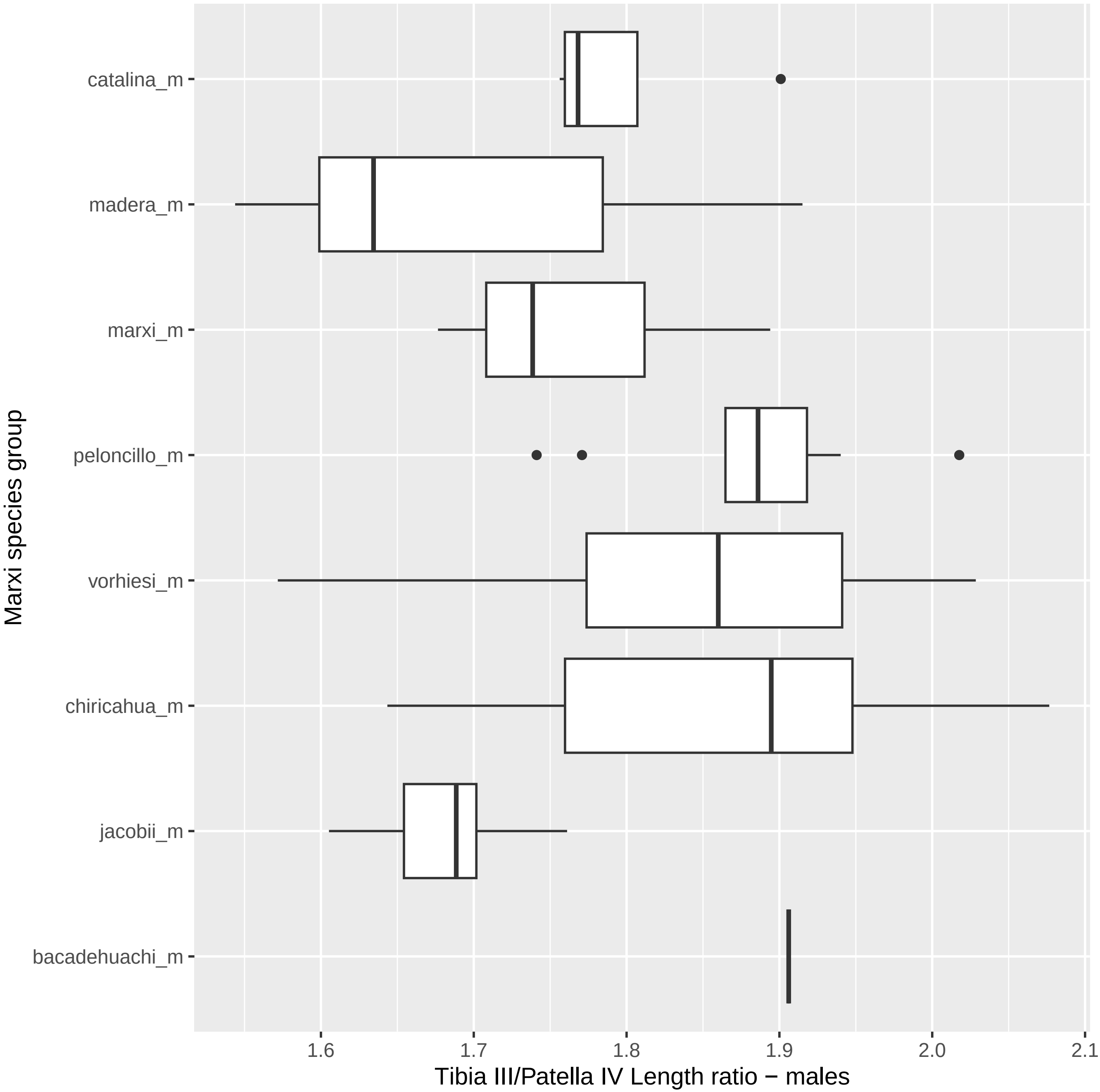

catalina_m
madera_m
marxi_m
peloncillo_m
vorhiesi_m
chiricahua_m
jacobii_m
bacadehuachi_m
Marxi species group
1.6
1.7
1.8
1.9
2.0
2.1
Tibia III/Patella IV Length ratio − males
